# Pearson’s Principle-Inspired Robust 2D Amorphous Ni-Fe-Co Ternary Hydroxides on Carbon Textile for High-Performance Electrocatalytic Water Splitting

**DOI:** 10.3390/nano12142416

**Published:** 2022-07-14

**Authors:** Rong Hu, Huiyu Jiang, Jinglin Xian, Shiyun Mi, Liyun Wei, Guangyu Fang, Jiayue Guo, Siqi Xu, Ziyang Liu, Huanyu Jin, Huimin Yu, Jun Wan

**Affiliations:** 1 State Key Laboratory of New Textile Materials and Advanced Processing Technologies, Hubei Key Laboratory of Biomass Fibers and Eco-Dyeing & Finishing, Wuhan Textile University, Wuhan 430200, China; fulonlon@163.com (R.H.); huiyujiang@wtu.edu.cn (H.J.); xianjinglin1006@163.com (J.X.); mishiyun0426@163.com (S.M.); wly9520ft@163.com (L.W.); mmyz1031@163.com (G.F.); 2105250704@mail.wtu.edu.cn (J.G.); 1905250434@mail.wtu.edu.cn (S.X.); lzy1341969071@163.com (Z.L.); 2 Institute for Sustainability, Energy and Resources, The University of Adelaide, Adelaide, SA 5005, Australia; 3 Future Industries Institute, University of South Australia, Mawson Lakes Campus, Adelaide, SA 5095, Australia

**Keywords:** layered hydroxide, ternary, amorphous, Pearson’s principle, electrocatalytic water splitting

## Abstract

Layered double hydroxide (LDH) is widely used in electrocatalytic water splitting due to its good structural tunability, high intrinsic activity, and mild synthesis conditions, especially for flexible fiber-based catalysts. However, the poor stability of the interface between LDH and flexible carbon textile prepared by hydrothermal and electrodeposition methods greatly affects its active area and cyclic stability during deformation. Here, we report a salt-template-assisted method for the growth of two-dimensional (2D) amorphous ternary LDH based on dip-rolling technology. The robust and high-dimensional structure constructed by salt-template and fiber could achieve a carbon textile-based water splitting catalyst with high loading, strong catalytic activity, and good stability. The prepared 2D NiFeCo-LDH/CF electrode showed overpotentials of 220 mV and 151 mV in oxygen evolution and hydrogen evolution reactions, respectively, and simultaneously had no significant performance decrease after 100 consecutive bendings. This work provides a new strategy for efficiently designing robust, high-performance LDH on flexible fibers, which may have great potential in commercial applications.

## 1. Introduction

Electrocatalytic water splitting is an economical and clean method for obtaining new energy, which consists of a hydrogen evolution reaction (HER) and oxygen evolution reaction (OER) [1]. Low-cost non-metal and non-noble metal catalysts have become the alternatives to catalysts, especially for layered double hydroxide (LDH) [2]. Although LDH has excellent intrinsic catalytic activity, its low conductivity will seriously affect electron transfer and reduce catalytic activity and stability [3,4,5], even in the widely used binary catalytic systems (such as NiFe-, NiCo-, CoFe-, CoMn-LDHs, etc.) [6,7,8,9]. It is reported that the electronic structure of a ternary solid solution can be further improved by introducing Co, V, or Mo elements on the basis of a binary catalyst [10,11,12]. By enhancing the synergistic effect between the newly added metal ions and the main metal in the lamellar network, ternary LDH with high electrocatalytic activity and good electrical conductivity can be formed [13,14]. For example, Duan et al. verified the influence of Co-doping on the performance of NiFe-LDHs through theoretical calculations and experiments [15]. The initial overpotentials of Co^3+^-doped NiFe-LDHs and Co^2+^-doped NiFe-LDHs were 33 mV and 18 mV, respectively, which were lower than that of the original NiFe-LDHs. In addition, the ternary configuration had an amorphous structure with abundant suspended bonds, coordination of unsaturated atoms, defects, and inherent disordered structure, which is beneficial to the charge transfer and ion diffusion of LDH and greatly improves its electrocatalytic performance [16]. Amal et al. reported a highly efficient and stable bifunctional electrocatalyst constructed from FeCoNi ternary hydroxide nanosheets with atomic thickness [17]; therefore, the optimal design of two-dimensional (2D) amorphous ternary LDH is the key to commercializing its high-performance water splitting electrocatalyst.

Actually, electrocatalysts are usually constructed by adding conductive agents and binders together in practical commercial applications. However, long-term service will lead to inevitable agglomeration, which greatly affects the service life of the catalyst [18]. The addition of non-conductive agents can be reduced to a large extent by using three-dimensional flexible self-supported electrodes. Carbon fiber (CF) with a high specific surface area and excellent mechanical flexibility has been proven to be the ideal candidate for the construction of high-performance flexible catalyst substrates [19,20,21]. At present, the combination methods of LDH and CF are mainly realized by hydrothermal and electrodeposition methods. Unfortunately, inert CF surfaces and limited deposition areas will lead to unstable LDH/CF interface bonding and low loading. It greatly affects the actual active area and cyclic stability of LDH during flexible deformation [22,23]; therefore, it is urgent to develop a new interface growth strategy with high loading, high activity, and good stability between LDH and CF.

Template-confined synthesis is a technique for growing nanomaterials by using the specific spatial limits of templates [24]. By constructing the composite structure of CF and templates, the interface bonding problem of CF and LDH can be transferred to the LDH growth mechanism on the template interface. Meanwhile, according to the Pearson’s Hard and Soft Acid−Base (HSAB) principle, a soft Lewis base can form stable complex with soft acid, which provides the possibility of interface design with high loading and good stability [25]. For instance, Guo et al. successfully prepared highly active and stable Ni(OH)_2_ using Cu_2_O as a sacrifice template and S_2_O_3_^2−^ as a coordination etch agent [26,27]. The strategy of combining the template/CF interface with the LDH/CF interface is expected to achieve the preparation of 2D amorphous ternary LDH flexible catalysts with high loading, high performance, and good stability.

Herein, we reported a method of the salt-template-assisted growth of robust 2D amorphous ternary LDH. The selected Cu_2_O template can be stably anchored on a CF surface by textile dip-padding technology. Meanwhile, based on the Pearson’s HSAB principle, a 2D amorphous NiFeCo ternary LDH with high loading, strong catalytic activity, and good stability can be constructed on the CF interface using Cu_2_O as a sacrificial template. The overpotential and Tafel slope of the prepared 2D amorphous ternary NiFeCo-LDH/CF electrode are 220 mV and 58 mV dec^−1^ in the OER and 151 mV and 67 mV dec^−1^ in the HER, respectively. The stable interfacial structure enables it to maintain high catalytic performance even after 100 repeats of continuous bending deformation. This Pearson’s Principle-inspired strategy is expected to provide a new idea for the commercial preparation of high-performance multivariate LDH and the highly stable construction of 2D materials at fiber interfaces.

## 2. Materials and Methods

### 2.1. Materials

CuCl_2_·2H_2_O (AR CAS:10125-13-0) and ascorbic acid (AR CAS: 50-81-7), NaOH (AR CAS: 310-76-2), SDS (AR CAS: 151-21-3), CoCl_2_·6H_2_O (AR CAS: 7791-13-1), and NiCl_2_·6H_2_O (AR CAS:7791-20-0) were purchased from Sinopharm Chemical Reagent Co. Ltd. in Shanghai, China. Mallinckrodt provided FeCl_2_·H_2_O (CAS: 13478-10-9). Without additional purification, all compounds were utilized as supplied. 

### 2.2. Preparation of Cu_2_O Nanocrystal

To begin, 0.85 g CuCl_2_·2H_2_O was dissolved in 500 mL water until the solution was blue and clear in hue. Then, 50 mL 2.0 M NaOH aqueous solution was added to the aforementioned solution. The hue of the solution progressively changed from blue-green to dark brown over time. Following that, a turbid red suspension was formed by adding 50 mL 0.6 M ascorbic acid solution to the mixture drop by drop under agitation. After treatment in an oil bath at 55 °C, the products were cleaned with deionized water and anhydrous ethanol 3 times, and then placed in a 60 °C vacuum oven to dry overnight.

### 2.3. Preparation of NiFeCo-LDH

A 300 mg Cu_2_O template was added to a 500 mL ethanol and aqueous solution. Then, 1.5 g PVP (MW = 30,000) was added and stirred for 10 min. A 10 cm × 10 cm carbon cloth was placed into soaking for 1 min and dip-padded once with a rolling car (repeated dip-padding twice). A carbon cloth loaded with Cu_2_O was obtained after vacuum drying at 70 °C. Then, a 2 cm × 2 cm carbon cloth was taken from it and added to 20 mL of an ethanol/water mixed solvent containing 1 mg CoCl_2_, 1 mg FeCl_2_, and 1 mg NiCl_2_. After soaking for 10 min, a certain amount of Na_2_S_2_O_3_ aqueous solution was added. Ternary NiFeCo hydroxide was prepared after a period of reaction at room temperature.

### 2.4. Electrochemical Measurements

A typical three-electrode setup with a CHI Electrochemical Station was used to evaluate electrochemical measurements (model CHI660E, Shanghai Chenhua Technology Co., Ltd., Shanghai, China). The reference electrode was a saturated calomel electrode (SCE) (0.5 M H_2_SO_4_) and Hg/HgO (1 M KOH), whereas the counter electrode was a carbon rod. To acquire the polarization curves, linear sweep voltammetry was performed at room temperature at a scan rate of 5 mV s^−1^, with an Ar flow maintained throughout the scanning procedure. The double layer capacitance (C_dl_) of the samples was used to calculate the electrochemical surface area (ESCA). The C_dl_ was calculated using a straightforward cyclic voltammetry (CV) approach. The CV was performed in a potential window at scan speeds ranging from 20 to 200 mV s^−1^. The twelve capacitive current (j_anodic_-j_cthodic_) at 0.575 V vs. RHE was plotted against various scan rates, while the slope obtained was divided by two to obtain the C_dl_ value. EIS was performed at the overpotentials with frequencies from 0.1 to 10^5^ Hz and an amplitude of 10 mV. Long durability tests were performed at their overpotentials.

## 3. Results

### 3.1. Mechanism for the Pearson’s Principle-Inspired Amorphous NiFeCo-LDH/CF

**Figure 1:**
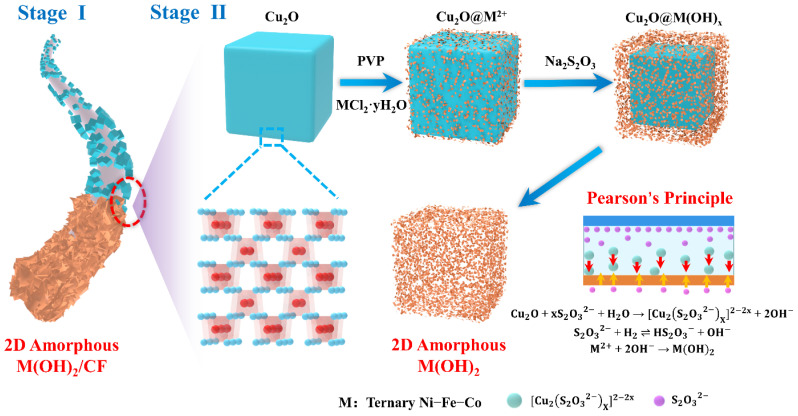
Two-dimensional (2D) amorphous M(OH)_2_ growth mechanism based on Pearson’s HSAB Principle.

The growth process of amorphous NiFeCo-LDH/CF is mainly divided into two stages: Stage Ⅰ, the stable anchoring of the Cu_2_O salt-template on the CF surface, and Stage Ⅱ, the growth of amorphous LDH on the Cu_2_O template sacrifice. Figure 1 is a schematic diagram of the growth mechanism. In Stage Ⅰ, it is difficult for the active material to directly grow stably on the interface of CF due to its inherent inertia. However, the Cu_2_O template can be stably anchored on the surface of CF by roller-pressing and vacuum-heating in the process of textile dip-padding, and the oxidation of Cu_2_O can be avoided. This stable template/CF interface architecture provides a guarantee for subsequent templates to sacrifice the growth of stable amorphous NiFeCo-LDH, and the cubic Cu_2_O templates also lay a foundation for the high-load construction of continuous growth. In Stage Ⅱ, according to Pearson’s HSAB Principle [25,26,27], the soft Lewis base can form stable complexes with the soft acid, while the hard base prefers the hard acid. The Cu^2+^ in the Cu_2_O template has the characteristics of soft acid, so the soft base ligands (S_2_O_3_^2−^, CN, SCN, etc.) should be selected as etchants. However, due to the high affinity between CN and metal ions and the insolubility of CuSCN, both ligands are not conducive to the formation of hydroxides. S_2_O_3_^2−^ is the best choice for a Cu_2_O etching agent. Its main functions are as follows:

(1) $${\mathrm{Cu}}_{2}O+{\mathrm{xS}}_{2}O_{3}{}^{2-}+{\;H}_{2}O\;\to {\left[{\mathrm{Cu}}_{2}{\left(S_{2}O_{3}{}^{2-}\right)}_{x}\right]}^{2-2x}+2{\mathrm{OH}}^{-}$$

(2) $$S_{2}O_{3}{}^{2-}+{\;H}_{2}O\;⇌{\;\mathrm{HS}}_{2}O_{3}{}^{-}+{\;\mathrm{OH}}^{-}$$

(3) $$M^{2+}+2{\mathrm{OH}}^{-}\;\to M{\left(\mathrm{OH}\right)}_{2}$$

M refers to the corresponding transition metal atom, M^2+^ refers to the metal ion, and M(OH)_2_ refers to the corresponding metal hydroxide. The role of S_2_O_3_^2−^ species is mainly shown in: (1) Synergistic etching occurs on Cu_2_O surface. Cu^2+^ and S_2_O_3_^2−^ form soluble ${\left[{\mathrm{Cu}}_{2}{\left(S_{2}O_{3}{}^{2-}\right)}_{x}\right]}^{2-2x}$ due to the soft−soft interaction. (2) Because of the unstable binding of Borderline Acid−Soft Base ($M^{2+}$ − $S_{2}O_{3}{}^{2-}$), other metal ions exist almost in a free state. (3) Cu_2_O co-etched and S_2_O_3_^2−^ hydrolyzed, which jointly promoted the formation of M(OH)_2_. Because of the highest concentration of ${\mathrm{OH}}^{-}$ at the etching interface, M(OH)_2_ is deposited at the interface first, which causes the generated hydroxides to imitate the geometry of the Cu_2_O template. It should be noted that the size of M(OH)_2_ will continue to grow as the reaction progresses until the metal ion concentration is reduced to a value that is not sufficient for precipitation.

### 3.2. Characterizations of Amorphous NiFeCo-LDH/CF

**Figure 2:**
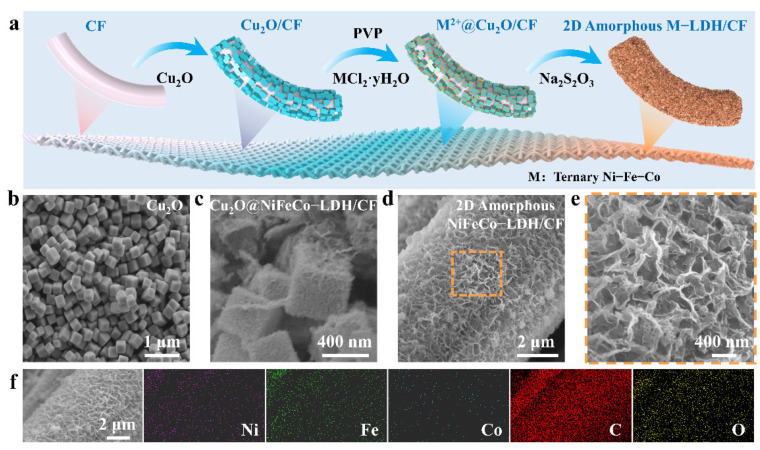
Preparation and morphology characterization of NiFeCo-LDH. (**a**) The general preparation process of NiFeCo-LDH. (**b**–**d**) SEM images of Cu_2_O, Cu_2_O@NiFeCo-LDH/CF, and NiFeCo-LDH/CF. (**e**) Partial enlargement of NiFeCo-LDH/CF. (**f**) The element distribution of NiFeCo-LDH/CF.

Based on the above mechanism, we designed the experimental process as shown in Figure 2a. Firstly, the cubic Cu_2_O was evenly dispersed in the mixed solvent of ethanol and water, while using the process of double dip-padding to fix it on CF. Then, PVP and metal chloride (nickel chloride, cobalt chloride, ferrous chloride) were added. These metal ions exist in the free state of the reaction system, and PVP can slow down the synergistic etching–precipitation rate and improve the quality of M(OH)_2_. Finally, Na_2_S_2_O_3_ was added to make Cu_2_O co-etch, accompanied by the formation of amorphous M(OH)_2_ and the load of M(OH)_2_ was realized on the fiber surface. Subsequently, the morphology and structure of the product were characterized. As shown in Figure 2b, the sample presents an obvious cubic shape with uniform particle distribution and a side length of about 400 nm. Figure 2c shows that the 2D lamellar structure grew uniformly on the surface of the cubic particle, which is similar to the core–shell structure formed on the surface of the cube. This structure corresponds to the above-mentioned co-etch deposition theory. Furthermore, scanning electron microscopy (SEM) images show that the cubic structure is completely etched and forms a network structure firmly wrapped on the fiber surface (Figure 2d). The SEM magnifying selection shows that the network structure is composed of interlacing connections of ordered two-position sheet structures (Figure 2e). This 2D structure can expose more active centers. Meanwhile, the long-range ordered structure increases the charge transfer and promotes the improvement in electrocatalytic performance. The spatial distribution of elements in the NiFeCo-LDH/CF structure is represented by energy-dispersive X-ray spectroscopy (EDS) mapping. Figure 2f shows the component elements of Ni, Fe, Co, C, and O, clearly showing the uniform composition profile of Ni, Fe, and Co in NiFeCo-LDH/CF.

**Figure 3:**
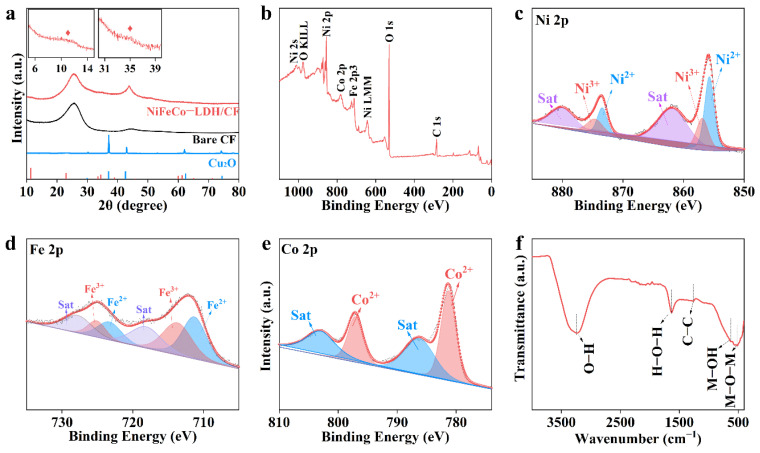
(**a**) XRD spectra of Cu_2_O and NiFeCo-LDH/CF. XPS spectra of NiFeCo-LDH/CF (**b**) survey, (**c**) Ni 2p, (**d**) Fe 2p, and (**e**) Co 2p. (**f**) FT-IR spectra of NiFeCo-LDH/CF.

X-ray diffraction (XRD) patterns are used to analyze the crystal structure changes of samples during the reaction process. As shown in Figure 3a, the diffraction characteristic peaks located at 29.9°, 37.0°, 42.5°, 62.4°, and 74.4° in the XRD diffraction pattern well correspond to the cubic phase Cu_2_O (PDF: 00–005–0667) [28], which proves that the cubic particles with uniform morphology are successfully synthesized cuprous oxides. The XRD pattern of NiFeCo-LDH/CF is similar to that of pure CF. There is no obvious diffraction peak, indicating that the prepared sample presents an amorphous nature [29]. This disordered structure and irregular surface provide a high degree of activity and exposure to abundant active sites. The amorphous state can also promote good mass transfer and provide more stable performance [30]. The X-ray photoelectron spectrometer (XPS) test was used to verify the chemical composition of NiFeCo-LDH/CF. The presence of Ni, Fe, Co, O, and C in NiFeCo-LDH/CF is further confirmed by the wide-sweep measurement spectrum shown in Figure 3b. The Ni 2p spectra show two peak binding energies of 873.1 eV and 855.9 eV, corresponding to Ni 2p_1/2_ and Ni 2p_3/2_, respectively, which are attributed to the Ni^2+^ oxidation state (Figure 3c). Additionally, two peaks were observed at 875.2 eV and 857.6 eV, respectively, indicating the presence of Ni^3+^ [31]. The spectra of Fe 2p show two peaks at 711.7 eV and 724.3 eV, respectively, belonging to Fe 2p_3/2_ and Fe 2p_1/2_ (Figure 3d). The peak value of Fe 2p_3/2_ can be divided into 710.6 eV and 713.3 eV and are assigned to Fe^2+^-OH and Fe^3+^-OH bonds, respectively [32]. Figure 3e shows two peaks at 781.2 eV (Co 2p_3/2_) and 796.9 eV (Co 2p_1/2_) in the Co 2p spectra, consistent with the Co^2+^ [33]. Furthermore, the Fourier-transform infrared (FT-IR) spectra of NiFeCo-LDH/CF are shown in Figure 3f. The wide peaks centered at 3445 cm^−1^ and 1634 cm^−1^ are assigned to the O-H stretching vibration of interlayer water molecules [34], and the skeleton vibration peak of the carbon ring in the NiFeCo-LDH/CF sample is located at 1546 cm^−1^. This is further evidence that graphene exists. The absorption peaks at 680 cm^−1^ and 510 cm^−1^ are derived from the vibration of the metal-O bond and the metal-O-metal bond, respectively [35].

### 3.3. Evaluation of HER and OER Performance

**Figure 4:**
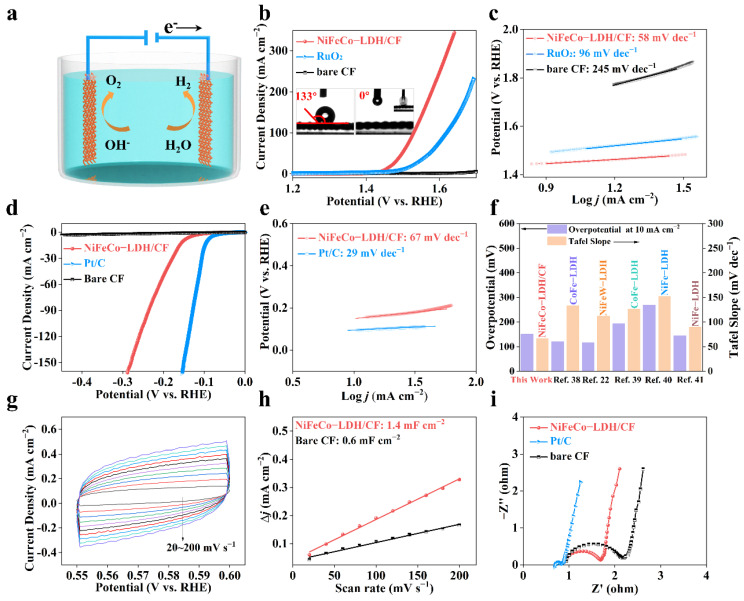
(**a**) Schematic diagram of electrocatalytic water splitting. (**b**) Oxygen evolution reaction (OER) performance of commercial RuO_2_, bare CF, and NiFeCo-LDH/CF in 1.0 M KOH. The contact angles of bare CF and NiFeCo-LDH/CF are illustrated. (**c**) Corresponding Tafel plots. (**d**,**e**) The LSV curves and Tafel slope of Pt/C bare CF and NiFeCo-LDH/CF. (**f**) Comparison of HER performance in common LDH. (**g**) Cyclic voltammograms of NiFeCo-LDH/CF. (**h**) Estimation of C_dl_ by plotting the current density variation against scan rate to fit a linear regression. (**i**) Nyquist plots of the above electrocatalysts.

Water splitting separation consists of two half-cell reactions: HER at the cathode and OER at the anode (Figure 4a). In order to evaluate the electrocatalytic performance of NiFeCo-LDH, a Hg/HgO electrode and graphite rod were used as the reference electrode and counter electrode, respectively. The catalytic performance of NiFeCo-LDH/CF was investigated in a 1 M KOH aqueous solution saturated with Ar using a typical three-electrode system. As shown in Figure 4b, when the current density of OER was 10 mA cm^−2^, the overpotential of NiFeCo-LDH/CF was 220 mV, which is better than that of commercial RuO_2_ (270 mV) and far better than that of original carbon cloth. It indicates that the 2D NiFeCo-LDH is the main source of electrocatalytic activity. Meanwhile, the illustrated contact angles of 133° on CF and 0° on NiFeCo-LDH/CF indicate that the morphology of the nanostructure improves the solvent wettability and leads to a superhydrophilic surface. Superhydrophilicity greatly improves the kinetics by increasing the contact area between the catalyst and H_2_O and promotes the performance of electrocatalytic water splitting [36]. The Tafel slope obtained by the LSV curve in Figure 4c is calculated according to the Tafel equation: *η* = b log *j* +a (where *η* represents overpotential, b represents the Tafel slope, and *j* represents the current density) [37]. NiFeCo-LDH/CF has a lower Tafel slope (58 mV dec^−1^) than those of the commercial RuO_2_ (96 mV dec^−1^) and pure carbon cloth (245 mV dec^−1^). The high conductivity and hydrophilicity of NiFeCo-LDH/CF promote the reaction kinetics. Subsequently, HER activity of NiFeCo-LDHs/CF was investigated in a 1 M KOH aqueous solution saturated with Ar. As shown in Figure 4d, NiFeCo-LDH/CF shows excellent HER activity with an overpotential of 151 mV at a current density of 10 mA cm^−2^. The low Tafel slope (67 mV dec^−1^) indicates that the ternary synergistic effect of Ni-Fe-Co in NiFeCo-LDH/CF, and the high electrical conductivity of carbon fiber, greatly promote the dynamic performance [14,35]. It is worth noting that the electrocatalytic water splitting performance of NiFeCo-LDH/CF is superior to many LDH catalysts at present (Figure 4f) [22,33,38,39,40,41,42,43,44,45,46,47,48,49,50].

The activity of the catalytic reaction is closely related to the exposed active area. Larger electrochemical active areas (ECSAs) can provide more reaction sites for catalytic reactions. ECSA can be calculated using the following formula: ESCA = C_dl_/Cs, where C_dl_ is an electrochemical double-layer capacitor [51]. C_dl_ was measured by cyclic voltammetry in the 0.55–0.6 V vs RHE range of scanning rates from 20 mV s^−1^ to 200 mV s^−1^, without a Faraday current (Figure 4g). As shown in Figure 4h, C_dl_ of NiFeCo-LDH/CF was 1.4 mF cm^−2^, much higher than 0.6 mF cm^−2^ of bare CF. It indicates that the NiFeCo-LDH/CF structure exposed more active sites, which contributed to enhancing the electrocatalytic activity. Meanwhile, the performance of catalysts is also closely related to the conductivity. To explore the electron transport capability, EIS was measured in an Ar-saturated aqueous solution of 1 M KOH [52]. Figure 4i shows that NiFeCo-LDH/CF has the smallest charge transfer resistance compared with the original carbon cloth, which means good reaction kinetics. Its good catalytic activity is attributed to the higher active sites exposed by this long-range interwoven 2D structure, as well as the good electrical conductivity and the synergistic effect of Ni-Fe-Co [18,53]. In addition, we further tested the electrocatalytic hydrogen evolution reaction (HER) performance of monadic and binary metal hydroxides, and their performance is shown in Figures S1 and S2. It can be seen that 3D transition metal base hydroxides, such as Ni, Co, and Fe, have quite high activity, since Fe ^3+^ can broaden the interlaminar space of 2D materials and enhance their mass transfer behavior [54]. Meanwhile, the synergistic effect between Fe and the LDH lamellar structure can shorten the ion transport distance at the nanometer scale, so that Fe-doping can further improve its catalytic performance. Moreover, Co-doping can accelerate the *OOH formation step of NiFe-LDH and reduce the overpotential of the deprotic step of NiFe-LDH [15], which further indicates that ternary NiFeCo-LDH has excellent catalytic performance in the HER process.

**Figure 5:**
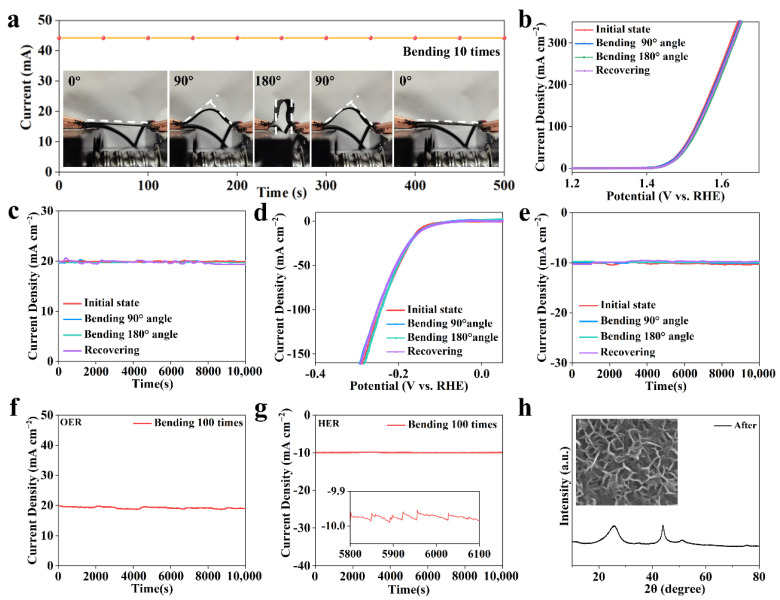
The mechanical flexibility of NiFeCo-LDH/CF. (**a**) I–t curve of the NiFeCo-LDH/CF bent with various curvatures under a constant voltage of 1 V. (**b**) Oxygen evolution reaction (OER) performance of NiFeCo-LDH/CF with various bending states, and (**c**) corresponding long-term stability testing. (**d**,**e**) Hydrogen evolution reaction performance of NiFeCo-LDH/CF. The long-term stability testing of NiFeCo-LDH/CF after bending 100 times, (**f**) OER, and (**g**) HER (the inset shows a magnified view of local current density). (**h**) The morphology and structure of the NiFeCo-LDH/CF after the long-term stability testing.

Generally, the interface between the active material and flexible substrate is unstable for flexible catalysts. It is speculated that this tight and stable NiFeCo-LDH/CF interface may be beneficial for improving its service life during deformation. We tested the cyclic stability of NiFeCo-LDH/CF under different bending deformations. Figure 5a shows the current stability of NiFeCo-LDH/CF in motion [55], which is continuously bent and recovered from 0° to 180° at a fixed potential of 1 V, with each cycle time being 50 s. It can be seen that the current hardly changes in the process of bending and recovering 10 times, showing good bending stability. We then tested its electrocatalytic properties in different bending states. As shown in Figure 5b, the OER performance was almost the same under different deformation states, and it also showed excellent long-term stability at a current density of 20 mA cm^−2^ (Figure 5c). Similarly, the stability of HER performance was also excellent (Figure 5d–e). Furthermore, we explored its bending resistance. After 100 bending recovery repeats, we tested its long-term stability, and its current density hardly changed after 100 bending repeats (Figure 5f–g). The illustration in Figure 5g shows the current change caused by bubble detachment. It shows that the superhydrophilic structure can increase the contact area between the catalyst and H_2_O and promote the electrocatalytic cracking water process from kinetics. Finally, the stabilities of the structure and morphology were characterized by XRD and SEM (Figure 5h). After the long-term stability test of NiFeCo-LDH/CF, both morphology and structure were also maintained, which further indicates that the NiFeCo-LDH/CF prepared by this method has excellent stability.

## 4. Conclusions

In summary, we successfully prepared 2D amorphous ternary NiFeCo-LDH/CF by using the salt-template-assisted method. The Cu_2_O template can be stably anchored on a CF surface by textile dip-padding technology. Subsequently, based on the Pearson’s HSAB Principle, the amorphous NiFeCo-LDH grew on the template surface through a synergistic corrosion–precipitation process. Compared with traditional methods, this unique growth mode was more conducive to obtaining amorphous LDH flexible catalysts with high loading, strong catalytic activity, and good stability. The prepared NiFeCo-LDH/CF electrode could not only show the overpotential of 220 mV and Tafel slope of 58 mV dec^−1^ in OER, but also show the overpotential of 151 mV and Tafel slope of 67 mV dec^−1^ in HER. Additionally, there was no significant reduction in catalytic performance, even after 100 repeats of continuous bending. This Pearson’s HSAB Principle-inspired strategy is expected to provide a new idea for the commercial preparation of high-performance LDH and the highly stable construction of 2D materials at fiber interfaces.

## Data Availability

The data presented in this study are available upon request from the corresponding authors.

## Supplementary Materials

The following supporting information can be downloaded at: https://www.mdpi.com/article/10.3390/nano12142416/s1, Figure S1: The LSV curves (a) and Tafel slope (b) of monohydroxide hydroxides; Figure S2: The LSV curves (a) and Tafel slope (b) of binary hydroxides; Table S1: Comparison of HER performance in coS1mmon LDH. References [22,33,38,39,40,41,42,43,44,45,46,47,48,49,50] are cited in the Supplementary Materials.
