# Supplementary material for: Pearson’s Principle-Inspired Robust 2D Amorphous Ni-Fe-Co Ternary Hydroxides on Carbon Textile for High-Performance Electrocatalytic Water Splitting
Source: Nanomaterials (Basel). 2022 Jul 14;12(14):2416. doi: 10.3390/nano12142416 (PMC9316908; doi:10.3390/nano12142416)
Supplement: Supplementary file 1 [file nanomaterials-12-02416-s001.zip › nanomaterials-1784234-supplementary.pdf]

nanomaterials

MDPI

Supplementary Materials

Pearson's Principle-Inspired Robust 2D Amorphous Ni-Fe-Co Ternary Hydroxides on Carbon Textile for High-Performance Electrocatalytic Water Splitting

Rong Hu [1,†], Huiyu Jiang [1,†], Jinglin Xian [1,†], Shiyun Mi [1], Liyun Wei [1], Guangyu Fang [1], Jiayue Guo [1], Siqi Xu [1], Ziyang Liu [1], Huanyu Jin [2,*], Huimin Yu [3,*] and Jun Wan [1,*]

[1] State Key Laboratory of New Textile Materials and Advanced Processing Technologies, Hubei Key Laboratory of Biomass Fibers and Eco-Dyeing & Finishing, Wuhan Textile University, Wuhan 430200, China; fulonlon@163.com (R.H.); huiyujiang@wtu.edu.cn (H.J.); xianjinglin1006@163.com (J.X.); mishiyun0426@163.com (S.M.); wly9520ft@163.com (L.W.); mmyz1031@163.com (G.F.); 2105250704@mail.wtu.edu.cn (J.G.); 1905250434@mail.wtu.edu.cn (S.X.); lzy1341969071@163.com (Z.L.)

[2] Institute for Sustainability, Energy and Resources, The University of Adelaide, Adelaide, SA 5005, Australia

[3] Future Industries Institute, University of South Australia, Mawson Lakes Campus, Adelaide, SA 5095, Australia

* Correspondence: huanyu.jin@adelaide.edu.au (H.J.); huimin.yu@unisa.edu.au (H.Y.); wanj@wtu.edu.cn (J.W.)

† These authors contributed equally to the work.

1. Figures S1 to S2

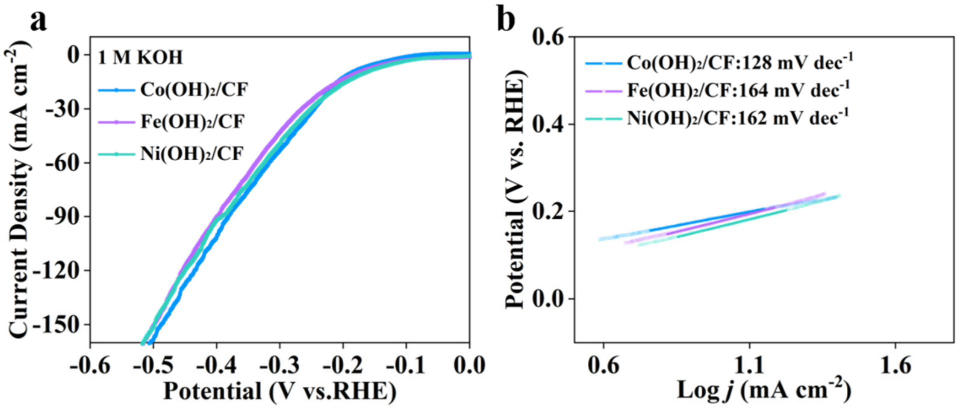

Figure S1. The LSV curves (**a**) and Tafel slope (**b**) of monohydroxide hydroxides.

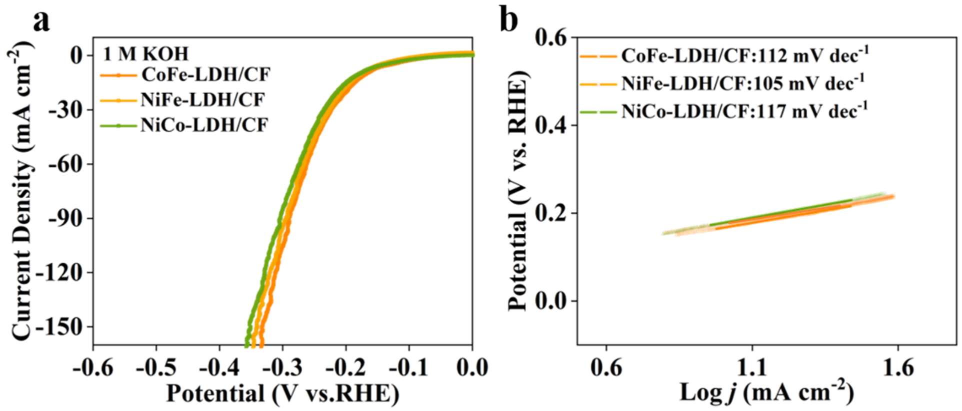

Figure S2. The LSV curves (**a**) and Tafel slope (**b**) of binary hydroxides.

2. Table S1

Table S1. Comparison of HER performance in common LDH.

Reference	Mateial	Electrolyte	η_{10} (mV vs.RHE)	Tafel slop (mV dec^{-1})
This Work	NiFeCo-LDH/CF	1 M KOH	151	67
Small 2019, 15, e1902551 [1]	CoFe LDH	1 M KOH	120	134
Electrochim. Acta 2021, 395. [2]	NiFeW-LDHs	1 M KOH	117	112
J. Mater. Chem. A 2020, 8, 2490-2497.[3]	CoFe LDH	1 M KOH	194	127
Adv Mater 2018, 30, 1706279.[4]	NiFe-LDH	1 M KOH	269	153
ACS Sustainable Chem. Eng. 2019, 7, 10035-10043.[5]	NiFe-LDHs	1 M KOH	145	90
Dalton Trans. 2017, 46, 8372-8376.[6]	NiCo-LDH	1 M KOH	130	141
J. Mater. Chem. A 2016, 4, 7245-7250.[7]	$Ni_{2.5}Co_{0.5}Fe$-OH	1 M KOH	110	93
ACS Appl. Energy Mater. 2018, 1, 1200-1209.[8]	CoFe LDH@g-C_3N_4	1 M KOH	210	79
Adv. Mater. 2017, 29, 1700017. [9]	NiFeLDH@DG	1 M KOH	270	110
Nanoscale 2018, 10, 19484-19491.[10]	NiFeLDH	1 M KOH	220	74
Energy Environ. Sci. 2019, 12, 572-581. [11]	NiFeLDH	1 M KOH	210	78
Small 2018, *14*, 1702568.[12]	CoFe-OH/Nifoam	1 M KOH	110	72
ACS Appl. Mater. Inter. 2018, *10*, 42453-42468.[13]	CoFe LDH with trace Fe	1 M KOH	170	83
ACS Appl. Mater. Inter. 2016, *8*, 34474-34481.[14]	Defected CoFe LDH	1 M KOH	300	95
Adv. Funct. Mater. 2018, *28*, 1804361.*[15]*	Au/ CoNi LDH	1 M KOH	210	92

3. Reference

1. Meng, X.; Han, J.; Lu, L.; Qiu, G.; Wang, Z.L.; Sun, C. Fe(2+) -Doped Layered Double (Ni, Fe) Hydroxides as Efficient Electrocatalysts for Water Splitting and Self-Powered Electrochemical Systems. *Small* **2019**, *15*, e1902551.
2. Ding, L.; Li, K.; Xie, Z.; Yang, G.; Yu, S.; Wang, W.; Cullen, D.A.; Yu, H.; Zhang, F. W-Induced Morphological Modification of NiFe Layered Double Hydroxides as Efficient Electrocatalysts for Overall Water Splitting. *Electrochim. Acta* **2021,** *395, 139199*.
3. Liu, S.; Zhu, J.; Sun, M.; Ma, Z.; Hu, K.; Nakajima, T.; Liu, X.; Schmuki, P.; Wang, L. Promoting the Hydrogen Evolution Reaction through Oxygen Vacancies and Phase Transformation Engineering on Layered Double Hydroxide Nanosheets. *J. Mater. Chem. A* **2020,** *8*, 2490-2497.
4. Chen, G.; Wang, T.; Zhang, J.; Liu, P.; Sun, H.; Zhuang, X.; Chen, M.; Feng, X. Accelerated Hydrogen Evolution Kinetics on NiFe-Layered Double Hydroxide Electrocatalysts by Tailoring Water Dissociation Active Sites. *Adv Mater* **2018,** *30*, 1706279.
5. Babar, P.; Lokhande, A.; Karade, V.; Pawar, B.; Gang, M.G.; Pawar, S.; Kim, J.H. Bifunctional 2D Electrocatalysts of Transition Metal Hydroxide Nanosheet Arrays for Water Splitting and Urea Electrolysis. *ACS Sustainable Chem. Eng.* **2019,** *7*, 10035-10043.

6. Liu, W.; Bao, J.; Guan, M.; Zhao, Y.; Lian, J.; Qiu, J.; Xu, L.; Huang, Y.; Qian, J.; Li, H. Nickel–Cobalt-Layered Double Hydroxide Nanosheet Arrays on Ni Foam as a Bifunctional Electrocatalyst for Overall Water Splitting. *Dalton Trans.* **2017,** *46*, 8372-8376.
7. Zhu, X.; Tang, C.; Wang, H. F.; Li, B. Q.; Zhang, Q.; Li, C.; Yang, C.; Wei, F. Monolithic-Structured Ternary Hydroxides as Free-standing Bifunctional Electrocatalysts for Overall Water Splitting. *J. Mater. Chem. A* **2016,** *4*, 7245-7250.
8. Bhowmik, T.; Kundu, M.K.; Barman, S. CoFe Layered Double Hydroxide Supported on Graphitic Carbon Nitrides: An Efficient and Durable Bifunctional Electrocatalyst for Oxygen Evolution and Hydrogen Evolution Reactions. *ACS Appl. Energy Mater.* **2018,** *1*, 1200-1209.
9. Jia, Y.; Zhang, L.; Gao, G.; Chen, H.; Wang, B.; Zhou, J.; Soo, M.T.; Hong, M.; Yan, X.; Qian, G.; Zou, J.; Du, A.; Yao, X. A Heterostructure Coupling of Exfoliated Ni–Fe Hydroxide Nanosheet and Defective Graphene as a Bifunctional Electrocatalyst for Overall Water Splitting. *Adv. Mater.* **2017,** *29*, 1700017.
10. Ye, W.; Fang, X.; Chen, X.; Yan, D. A Three-Dimensional Nickel–Chromium Layered Double Hydroxide Micro/Nanosheet Array as an Efficient and Stable Bifunctional Electrocatalyst for Overall Water Splitting. *Nanoscale* **2018,** *10*, 19484-19491.
11. Qiu, Z.; Tai, C. W.; Niklasson, G.A.; Edvinsson, T. Direct Observation of Active Catalyst Surface Phases and the Effect of Dynamic Self-Optimization in Nife-Layered Double Hydroxides for Alkaline Water Splitting. *Energy Environ. Sci.* **2019,** *12*, 572-581.
12. Babar, P.; Lokhande, A.; Shin, H.H.; Pawar, B.; Gang, M.G.; Pawar, S.; Kim, J.H. Cobalt Iron Hydroxide as a Precious Metal-Free Bifunctional Electrocatalyst for Efficient Overall Water Splitting. *Small* **2018,** *14*, 1702568.
13. Rajeshkhanna, G.; Singh, T.I.; Kim, N.H.; Lee, J.H. Remarkable Bifunctional Oxygen and Hydrogen Evolution Electrocatalytic Activities with Trace-Level Fe Doping in Ni- and Co-Layered Double Hydroxides for Overall Water-Splitting. *ACS Appl. Mater. Inter.* **2018,** *10*, 42453-42468.
14. Liu, P.F.; Yang, S.; Zhang, B.; Yang, H.G. Defect-Rich Ultrathin Cobalt–Iron Layered Double Hydroxide for Electrochemical Overall Water Splitting. *ACS Appl. Mater. Inter.* **2016,** *8*, 34474-34481.
15. Sultana, U.K.; Riches, J.D.; O'Mullane, A.P. Gold Doping in a Layered Co-Ni Hydroxide System Via Galvanic Replacement for Overall Electrochemical Water Splitting. *Adv. Funct. Mater.* **2018,** *28*, 1804361.
